# The focal alteration and causal connectivity in children with new-onset benign epilepsy with centrotemporal spikes

**DOI:** 10.1038/s41598-018-23336-z

**Published:** 2018-04-09

**Authors:** Sihan Chen, Jiajia Fang, Dongmei An, Fenglai Xiao, Deng Chen, Tao Chen, Dong Zhou, Ling Liu

**Affiliations:** 1 Epilepsy Center, Department of Neurology, West China Hospital, Sichuan University, Chengdu, PR China; 2 Department of Neurology, Fourth Affiliated Hospital, School of Medicine, Zhejiang University, Yiwu, PR China

## Abstract

The aim of the current study was to find the epileptic focus and examine its causal relationship to other brain regions in children with new-onset benign childhood epilepsy with centrotemporal spikes (BECTS). Resting-state functional magnetic resonance imaging (fMRI) was performed in 66 children with BECTS and 37 matched control children. We compared the amplitude of low frequency fluctuation (ALFF) signals between the two groups to find the potential epileptogenic zone (EZ), then used Granger causality analysis (GCA) to explore the causal effects of EZ on the whole brain. Children with BECTS had significantly increased ALFF in the right Broca’s area, and decreased ALFF in bilateral fusiform gyrus. The patients also showed increased driving effect from the EZ in Broca’s area to the right prefrontal lobe, and decreased effects to the frontal lobe and posterior parts of the language network. The causal effect on left Wernicke’s area negatively correlated with verbal IQ (VIQ) score. Our research on new-onset BECTS patients illustrates a possible compensatory mechanism in the language network at early stages of BECTS, and the negative correlation of GCA and VIQ suggest the disturbance of epileptiform activity on language. These findings shed light on the mechanisms of and language dysfunction in BECTS.

## Introduction

So-called “benign epilepsy with central-temporal spike” (BECTS) is the most common epilepsy syndrome among children with epilepsy^1^. It has been recognized as a self-limited benign epilepsy syndrome^2^, a categorization challenged by recent and accumulating evidence suggesting that children with BECTS may suffer from cognitive co-morbidities including language dysfunction^3,4^, attention deficit disorder, and memory problems^5–7^. Using resting-state functional magnetic resonance imaging (fMRI), which can depict temporal and spatial characteristics of brain activity^8–10^, researchers have now proposed a possible underlying mechanism for BECTS that includes the disruption of several high-order cognitive functional networks^11–18^. This includes network re-organizations (including intra- and inter-hemisphere shifts) in epilepsy patients that suggest a compensatory shift to right homologous brain areas^19,20^, or a flexible language network comprising dorsal and ventral circuits^21–23^. However, these studies described the involvement of large scale brain networks but did not provide evidence for a direct causal relationship between the involved areas, despite the recent intense interest in the dynamic characteristics of epilepsy networks^10^.

Broca’s and Wernicke’s areas play central roles in the language system. Recent resting-state fMRI studies revealed that the connection maps of these two key areas include short pathways to cingulate gyrus and inferior parietal cortex (BA 40), and long range connections to medial prefrontal lobe (BA11/10), angular gyrus, occipital cortex (BAs 17, 18, 19), temporal cortex (BAs 21 and 22), and sub-cortical regions including striatum and thalamus^24^. Wernicke’s area also shows more long-range and right-lateralized connections, demonstrating the role of Wernicke’s area in modulating multiple-language information and processing figurative language in contrast to processing literal interpretation^25–27^. Together, this work has begun to parse a possible framework for the language network as a whole.

Functional neuroimaging has produced a more detailed assessment of the language networks and their lateralization that is consistent with the results provided by the gold standard methods^28^. Amplitude of low frequency fluctuation (ALFF) signals can detect regional spontaneous activity by measuring the magnitude of spontaneous blood oxygenation level dependent (BOLD) signals, ultimately reflecting brain activity in a time series. In epilepsy patients ALFF is positively correlated with the number of epileptic discharges, and can thus be used for localizing epileptic foci in the mesial temporal lobe, other cortical and subcortical structures associated with cognitive impairment^29,30^, and possibly regional language networks^31^. Functional connectivity analyses have revealed reduced connectivity between the sensorimotor and language networks in children with BECTS^12,32^.

While static network models aim to describe the structures of brain networks^33,34^, dynamic causal modeling in fMRI implies causation and effective connectivity (directed effects from one region to another)^35^. Granger causality analysis (GCA) is used for inferring the direction of information flow in these brain networks^36,37^. One study on BECTS patients found an increased driving effect from the ROI zone to the right medial frontal cortex and posterior cingulate cortex and decreased causal effects to left inferior frontal gyrus^16^. That study was conducted with patients receiving antiepileptic drugs (AED), however, and AED treatment might interfere with local brain activity^38^. To eliminate the potential effects of AED and avoid the possible influence of long term aberrations in spikes on network connectivity^39^, here we conducted an fMRI study on new-onset treatment-naïve children within three months of their diagnosis with BECTS. We hypothesized that a comparison of the global ALFF signal between patients and healthy controls would find focal functional alterations in the key components of language networks and that the driving effect from the affected zone(s) would illustrate information flow alterations (intra- and inter-hemisphere) within the language framework.

## Results

### Neuropsychological testing

Table 1 shows the demographic, clinical and neuropsychological characteristics of the patients with BECTS and healthy controls. There was no significant difference between patients and control group in FSIQ or PIQ. The VIQ scores in the children with BECTS were slightly lower than the control group but this difference was not significant (t = 1.475, p = 0.143).

**Table 1:** Demographic and neuropsychological characteristics of BECTS patients and healthy controls.

Characteristic	Patients	Controls	t/χ^2^	P value
Age (years)	9.7 ± 2.1	9.4 ± 2.1	−0.723	0.471^a^
Sex (female/male)	28/38	17/20	0.120	0.73^b^
Handedness	60^R^/4^L^/2^A^	34^R^/3^L^	—	—
EEG lateralization	31^R^/28^L^/7^A^	—	—	—
FSIQ	98.4 ± 11.1	100.8 ± 11.8	1.031	0.305^a^
VIQ	102.2 ± 5.9	104.4 ± 8.8	1.475	0.143^a^
PIQ	96.4 ± 13.6	98.9 ± 10.4	0.983	0.328^a^

The intelligence quotient (IQ) scores in patients and controls were based on the results of 66 and 37 participants, respectively. R: right sided; L: left sided; A: ambidextrous; FSIQ = full scale IQ; VIQ = verbal IQ; PIQ = performance IQ; ^a^Two-sample t test; ^b^χ^2^ test.

### Between-group analysis of ALFF

**Figure 1:**
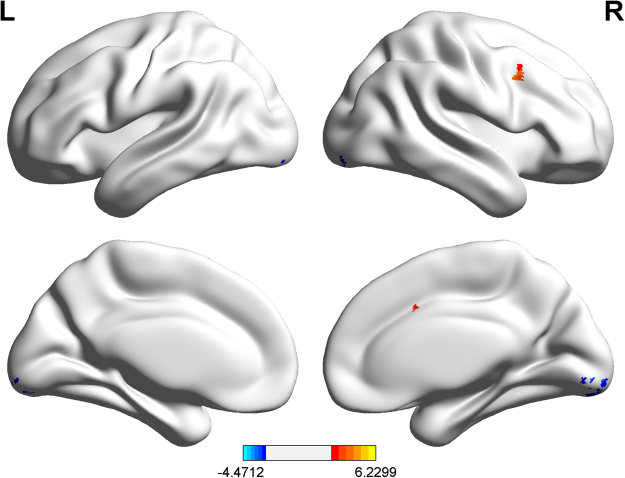
Brain regions showing abnormal ALFF in patients with BECTS. The warm (red) and cold (blue) colors represent higher and lower ALFF, respectively, in patients compared with controls (P < 0.05, GRF corrected). Color bar represents t values.

Compared to the healthy control participants, the patients with BECTS showed significantly increased ALFF in the right Broca’s area (t = 6.230, p < 0.05, GRF corrected, two tailed) and decreased ALFF at the bilateral fusiform gyrus (BA 18; t = −4.363, p < 0.05, GRF corrected, two tailed; Fig. 1).

### Voxel-wise GCA

#### Seed-to-whole-brain analysis

**Figure 2:**
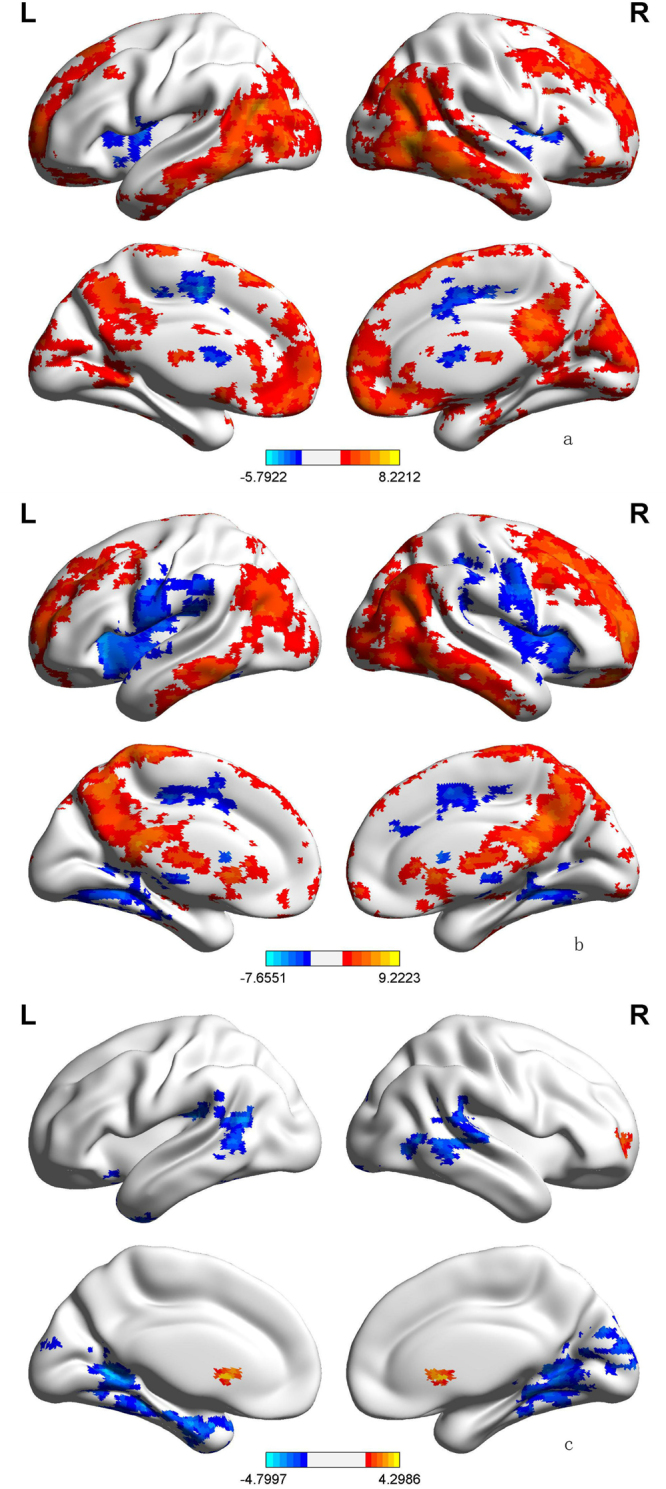
Voxel-wise GCA. (**a**) Regions showing significant causal effect with the seed in controls. (**b**) Regions showing significant causal effect with the seed in patients. (**c**) Regions showing abnormal causal effect with the seed in patients compared with controls. Color bar represents t values.

We chose to set the affected right Broca’s area as the seed zone for the analysis. In the seed-to-whole-brain analysis we identified a number of cortical areas and subcortical structures that were driven by the seed region in patients with BECTS (Fig. 2b). The pattern in the healthy controls is demonstrated in Fig. 2a. Compared to healthy controls, the patients showed an increased driving effect from the seed area to the caudate and prefrontal cortex (BA11), along with decreased effects on the bilateral lingual gyrus (BA18), right middle temporal gyrus (BA22), left angular gyrus (BA21) and left hippocampus (BA36; p < 0.05, GRF corrected, two tailed; Fig. 2c; details in Table 2).These results concur with the existing language framework.

**Table 2:** Regions showing abnormal causal effect with epileptogenic zone in patients (seed-to-whole-brain).

Brain region	MNI	BA	Peak t value	Control	BECTS
lingual gyrus(R)−	12, −50, 4	BA18	−3.840	3.471^※^	−0.252
lingual gyrus(L)−	−17, −56, 4	BA18	−4.005	2.634^※^	−2.849
Midtem gyrus(R)−	67, −42, 6	BA22	−3.63	4.753	0.4882
angular gyrus(L)−	−39, −33, 18	BA21	−4.388	2.314^※^	−4.189
Hippocampus(L)−	−23, −12, −25	BA36	−3.186	1.957^※^	−2.479
Caudate(R)+	20, 10, −21	NA	3.830	2.546	7.439
Prefrontal(R)+	25, 48, −2	BA11	3.442	−0.981^※^	4.299

BA = Brodmann’s area; L = left side; R = right side; MNI = Montreal Neurological Institute coordinate; Midtem: middle temporal gyrus; Prefrotal: pre-frontal lobe; NA: not available; +: increased causal effect from seed; −: decreased causal effect from seed; The last two columns show the t value of the corresponding peak voxel within the patient and control group, respectively. Values with “※” show that the mean causal effect of the corresponding cluster is significantly different from zero.

#### Whole-brain-to-seed analysis

Whole-brain-to-seed analysis showed that there was no abnormal positive or negative driving effect from whole brain to seed in the patients with BECTS.

### Correlation analysis

**Figure 3:**
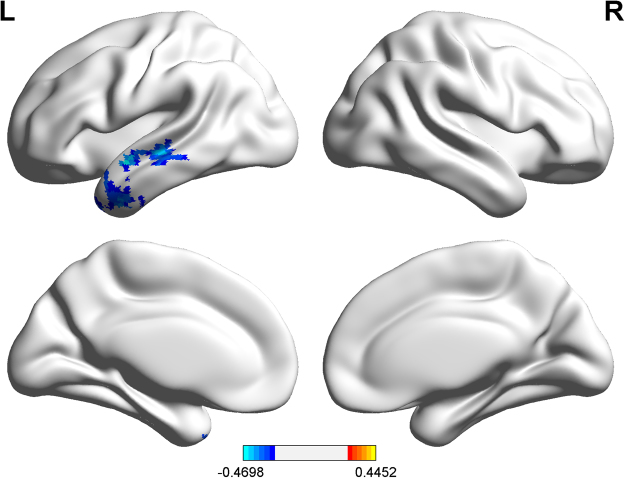
Correlation analysis: Negative correlation between GCA of Wernicke’s area and VIQ scores. Color bar represents t values.

We performed correlation analysis between the VIQ score and the GCA map of the patients (Fig. 3). This analysis revealed a negative correlation between VIQ score and the driving flow from right Broca’s area to the left Wernicke’s area (t = −0.451, p < 0.05, GRF corrected, two tailed).

## Discussion

This study on new-onset treatment-naïve BECT patients found increased local brain activity in the right Broca’s area. From that seed area, we then found increased causal effects to the caudate, thalamus, and prefrontal lobe, as well as decreased effects to the middle temporal lobe, angular gyrus, lingual gyrus, and hippocampus; all of which are within the known language network. We also found a negative correlation between VIQ scores and the driving effect to right Wernicke’s area. In contrast to some previous reports, we didn’t find any significant difference in IQ test between two groups.

We found that the patients showed higher ALFF signal in the right Broca’s area and decreased signal at bilateral fusiform gyrus. The classical neuropsychological model implies that language is a rigid and monolithic function^40^, however, more recently neuroimaging studies have broadened our perspective on language system^41^. Current opinion suggests that language processing is underpinned by a flexible cerebral network including areas beyond the classical language regions in both hemispheres^41,42^, and that the language system is based on both inter-hemispheric and intra-hemispheric connections^43^. In accordance with this view, the functional network in patients with focal epilepsy might shift from the pathological spike-affected areas to adjacent areas and homologues^19^. Converging evidence also suggests that chronic epileptic activity can result in a developmental shift of language processing from the left to the right hemisphere or event re-route language pathways from traditional to non-traditional areas^44^. The increased ALFF activity that we observed in the right Broca’s area may imply a compensatory role for the right Broca’s area at the beginning stages of the disease. This view is supported by findings of right homologue area recruitment during language tasks in focal epilepsy patients^22^. Research combining fMRI and DTI has also noted that the right Broca’s area serves as a hub in the long-distance dorsal pathway along the arcuate and superior longitudinal fascicle (AF/SLF) and as a key node in high-order cognitive processing^23^.

The decreased ALFF signal observed in fusiform gyrus might illustrate a down-regulation of peripheral components within the dynamic language processing network. The fusiform gyrus is part of the “visual word form area”in occipital lobe^45^, and together with other components, it plays a supporting role in speech and language processing^46^. In our study the deficit of fusiform gyrus recruitment might illustrate the impact of pathological spikes on language system and might underlie the language dysfunction noted in BECTS patients.

GCA analysis has previously been used in epilepsy patients to analyze the EEG and fMRI signal and it enables us to understand better how seizure activity initiates, propagates, and terminates, providing an overview of information flow within the epilepsy network^47,48^, though there is some controversy over how to improve its accuracy^49^. In our study the driving effect from the right Broca’s area illustrates that the flexible language system may be based on both core areas including key language components and peripheral areas such as the fusiform gyrus and the para-hippocampal gyrus; these are connected via inferior fronto-occipital fasciculus (IFOF), which serves as an important portion of ventral pathway^50^. One recent theoretical framework proposes that functional interactions between brain regions might change over time, and that the patterns of inter-regional communication may determine the degree of functional specialization for a given region^51^—that is, peripheral areas that are transiently engaged during linguistic processing may come to help complete the processing of language tasks^52,53^. This theory explains the involvement of posterior network areas including angular gyrus, fusiform gyrus, occipital lobe and mid-temporal gyrus in many high-order cognitive tasks including language^54^.

The increased ability for Broca’s area to drive frontal lobe coupled with its decreased driving effect on posterior areas may imply elevated information stream density in the frontal-parietal circuit (including frontal lobe and Broca’s area) and disrupted connections from core components to peripheral components of the language network including the occipital lobe, angular gyrus and others. Similar findings have been observed in new-onset BECT patients using the Regional Homogeneity (ReHo) approach^55^. This also concurs with other observations that the frontal lobe fails to deactivate task negative network components in epilepsy patients^56^. A study on children with focal epilepsy also found that the ventral language network might be more vulnerable under epilepsy conditions while the dorsal language network was relatively unaffected^57^. A study of subcortical electrical stimulation on the anterior part of the arcuate fascicle linking the inferior parietal and frontal cortices also showed that rostral components can regulate the language system^58^. The imbalance of the rostral-caudal components might be due to the disturbance of temporal segregation of auditory and visual perception and thus of speech comprehension and production, which may further underlie the potential language impairment in long-term epilepsy^24^.

Overall IQ performance did not differ significantly between the patients and the healthy volunteer. This is in accordance with previous findings in children with new-onset BECTS^59^, and may reflect compensation by the increased Broca-frontal connection observed. The short duration of epilepsy in BECTS patients may also contribute to the negative finding^60^. The negative correlation between VIQ and the GCA effect on left Wernicke’s area, however might illustrate the disturbance of the right Broca’s area. Previous studies have revealed the long-path of Wernicke’s area and its right-lateralized connection in the language network^24^. Researchers hypothesize that these connections help Wernicke’s area modulate multiple-language information^25–27^. The abnormal driving effect from right Broca’s area might thus interfere with VIQ performance.

One limitation of the current work is that a cross sectional cannot capture the longitudinal development of the delicate balance of the language network, which would likely provide more evidence of the reorganization and rebalance of these components over time. We also found no significant differences between patients and healthy controls on IQ scores. This could be explained by a compensatory mechanism, or could reflect the relatively limited number of patients represented in this sample. In the future we would like to enlarge the study size to address this question.

This study demonstrates for the first time the imbalance of the language system in the early stages of the disease and a possible rebalancing of the language system under these conditions. We found that children with new-onset BECTS illustrate an increased driving effect from the right Broca’s area to the frontal lobe and sub-cortical areas and decreased effects to the caudal parts of the flexible language network. The overall IQ scores did not differ significantly between the patients and healthy controls, but the driving effect from right Broca’s area to left Wernicke’s area does seem to interfere with VIQ performance, and these disruption may underlie future language dysfunction in these patients. A follow-up longitudinal study may better illustrate the natural history of BECTS and its comorbidities, which will deepen our knowledge of brain development under pathological conditions.

## Methods

### Participants

66 children suffering from BECTS (28 girls, mean age 9.7 ± 2.1.years, range 5–14 years) and 37 healthy controls (HC; 17 girls, mean age 9.4 ± 2.2 years, range 5–15 years) were included, all participants were attending public school. Patient diagnosis was based on the diagnostic criteria of the ILAE guidelines (ILAE, 1989). Exclusion criteria included: (i) root mean square head motion > 2 mm translation or 1.5° rotation; (ii) other neurological or psychiatric disease (including BECTS-associated co-morbidities such as ADHD and migraine); (iii) focal pathological MRI performance. (iv) Wechsler Intelligence Scale for Children China-Revised (WISC-CR) test score < 75.

We recruited patients from the epilepsy center of the neurology department at West China Hospital of Sichuan University. 60 patients were right-handed, 4 patients left-handed and 2 ambidextrous; 34 controls were right-handed, 3 left-handed. At the time of inclusion in the study, EEG spike foci were left-sided in 28 patients, right-sided in 31 and bilateral in 7, including 4 patients with left-side predominance. The demographic and clinical characteristics are presented in Table 1. Written informed consent was obtained from the parents or guardians of all subjects and the present study was approved by the local Ethics Committee of West China Hospital of Sichuan University. All experiments were performed in accordance with relevant guidelines and regulations.

### Neuropsychological testing

The Wechsler Intelligence Scale for Children China-Revised (WISC-CR) test was applied to evaluate intelligence and to generate Full Scale, Verbal, and Performance IQ scores (Table 1)^61^.

### Data acquisition

All resting-state functional MRI data were acquired on a 3 T magnetic resonance imaging system (Siemens Trio, Erlangen, Germany) within 3 months of diagnosis. The scan parameters were as follows: repetition time/echo time (TR/TE) = 2000/30 ms; flip angle = 90°; 30 axial slices per volume; 5 mm slice thickness (no slice gap); matrix = 64 × 64; FOV = 240 × 240 mm^2^; voxel size = 3.75 × 3.75 × 5 mm^3^. Each functional scan contained 200 image volumes. The participants were instructed not to think about anything in particular and to keep their eyes closed during the scan. Head motion was minimized using foam pads. A built-in camera was used to monitor subjects and check whether they fell asleep during the scan.

### fMRI data processing

Functional MRI image preprocessing was done on the toolbox for Data Processing & Analysis for Brain Imaging (DPABI; http://www.restfmri.net)^62^, which synthesizes procedures in the Resting State fMRI Data Analysis Toolkit (REST; http://www.restfmri.net)^63^ and Statistical Parametric Mapping (SPM8; www.fil.ion.ucl.ac.uk/spm). The first 10 images were removed to ensure steady-state longitudinal magnetization, and the remaining images were then corrected for temporal differences and head motion. After subject selection, neither translation nor rotation parameters exceeded ±2 mm or ±1.5°. The functional images were then registered into 3D T1 image of each participant at a resolution of 3 × 3 × 3 mm^3^. We then spatially smoothed the images with a 6 mm full-width half-maximum isotropic Gaussian kernel, to regress out nuisance signals (white matter, cerebrospinal fluid signals, and 6 head-motion parameters). Finally, linear trends were removed from the time courses and temporal band-pass filtering was performed (0.01–0.08 Hz)

### ALFF analysis

ALFF is the averaged square root of activity in the low-frequency band (0.01–0.08 Hz) [18]. The ALFF value of each voxel was standardized by dividing the value by the whole-brain mean ALFF value. Two-sample T tests were used to compare the differences in ALFF between the patients and control groups. Using the Gaussian Random Field theory correction (GRF) built into the REST software, we then applied a corrected significance level of voxel level p < 0.05 and cluster level p < 0.05 (GRF corrected, two-tailed).

### GCA

A cluster showing increased ALFF was identified in right Broca’s area, and the peak voxel (with a 3 mm radius) in this cluster was used as the seed region for the GCA analysis. The voxel-wise coefficient GCA was performed in the whole brain using REST-GCA, a REST software plug-in ref.^63^. We applied bivariate coefficient GCA to investigate the causal relation between the seed zone and each voxel in the entire brain. One-sample T tests were performed for the causal effects within each group, with an uncorrected significance level of p < 0.05. Two-sample T-tests were then performed on the causal effects between groups using the GRF corrected significance level of voxel level p < 0.05 and cluster level p < 0.05 (two-tailed). To explore whether the neuroimaging measures correlated with the disease features in BECTS children, Pearson’s correlation was performed between the causal effect map and VIQ scores at global range.
